# Treatment in the STAMPEDE era for castrate resistant prostate cancer in the UK: ongoing challenges and underappreciated clinical problems

**DOI:** 10.1186/s12885-018-4527-y

**Published:** 2018-06-19

**Authors:** Rosa U. Greasley, Rebecca Turner, Karen Collins, Janet Brown, Liam Bourke, Derek J. Rosario

**Affiliations:** 1 The Centre for Sport and Exercise Science, Faculty of Health and Wellbeing, Sheffield Hallam University, Sheffield, UK; 2 The Department of Oncology and Metabolism, University of Sheffield Medical School, Sheffield, UK; 3 Centre for Health and Social Care Research, Faculty of Health and Wellbeing, Sheffield Hallam University, Sheffield, UK; 4 Academic Unit of Clinical Oncology, Department of Oncology and Metabolism, University of Sheffield, Weston Park Hospital, Sheffield, UK

## Abstract

**Background:**

This study aimed to explore the opinions of healthcare professionals regarding the management of men with advanced prostate cancer with particular emphasis on treatment timing and sequencing; treatment adverse-effects and exercise a supportive therapy.

**Methods:**

Semi-structured interviews with a purposively selected group of healthcare professionals involved in prostate cancer care within the NHS, conducted over the phone or face to face. A total of 37 healthcare professionals participated in the interviews including urologists, clinical oncologists, medical oncologists, clinical nurse specialists, general practitioners, physiotherapists, exercise specialists, service managers, clinical commissioners and primary care physicians.

**Results:**

The availability of newer treatments for advanced prostate cancer as well as results from the STAMPEDE and CHAARTED trials has resulted in new challenges for patients and HCPs. This includes the impact of an increased workload on oncologists, a potential lack of clinical continuity between urology and oncology and uncertainties regarding optimal selection, timing and sequencing of chemotherapy and second-line treatment. Fitness for treatment in advanced prostate cancer populations remains a significant barrier to accessing therapies for patients with a poor performance status. Among this, muscle wastage can significantly affect performance status and consequentially compromise cancer therapy. Exercise was regarded as a potential therapy to mitigate the adverse-effects of treatment including the prevention or reduction in muscle wastage.

**Conclusions:**

There is a lack of data guiding clinicians in this post STAMPEDE and CHAARTED era, work is needed to reassess and optimize the prostate cancer care pathway as it evolves. Exercise should be explored as a therapeutic option to mitigate the effects of long term ADT. Further study from a wider cohort of both prostate cancer care specialists and patients will aid in establishing a highly functioning pathway with optimal individualised care.

**Trial registration:**

Sustained exercise TrAining for Men wIth prostate caNcer on Androgen deprivation: the STAMINA programme (RP-DG-1213-10,010). REC Reference: 15/SW/0260 IRAS Project ID: 178340 Hospital ID: STH 18391 approved on 24/08/2015.

**Electronic supplementary material:**

The online version of this article (10.1186/s12885-018-4527-y) contains supplementary material, which is available to authorized users.

## Background

Until 2010, docetaxel chemotherapy remained the only therapy for castrate resistant prostate cancer (CRPC) which demonstrated a significant survival benefit (18.9 months vs 16.5 months in the docetaxel groups vs mitoxantrone group) [1, 2]. Post 2010, there has been an introduction of five other therapeutic options which have also shown a survival benefit in phase III trials: cabazitaxel, sipuleucel-T, radium-223, abiraterone and enzalutamide [3–8]. Improvements in survival of men with the use of docetaxel at earlier (hormone sensitive) stages of metastatic (M1) prostate cancer have been demonstrated in the recent multicentre randomized controlled trials STAMPEDE and CHAARTED [9–11] .The introduction of docetaxel upon initiation of androgen deprivation therapy (ADT) had a significant survival benefit when compared to the ADT group alone in hormone-sensitive M1 disease (57.6 vs 44.0 months, 95% CI 0.47 to 0.81; *P* < 0.001) [9]. Consequentially, in 2015 changes in clinical practice followed and an increasing number of men will receive chemotherapy earlier in their prostate cancer care pathway.

This rapid growth in treatment options since 2010 and the uncertainty around the efficacy of newer agents in the post-docetaxel setting (due to earlier exposure to docetaxel) presents oncologists and urologists with issues concerning the optimal sequencing and adherence to subsequent treatment regimens as well as potential adverse-effects of cytotoxic agents and the impact on quality of life (QoL) [12].

Additionally, in the UK, urologists do not prescribe taxane-based chemotherapeutics for prostate cancer, and the optimal referral route between urology and oncology is variable between National Health Service (NHS) trusts. Establishing coherent and optimised care pathways not only offers obvious benefits for impacting treatment outcomes, but also creates a culture of ownership, responsibility and accountability within the clinical team [13].

There are unappreciated emerging needs common to advanced cancer patients which are not being adequately addressed in uro-oncology. The adverse-effects of long term ADT need to be explored to aid clinicians in treatment based decision making and direct research with an aim to reduce those effects that have the biggest detriment on the health and wellbeing of these men. This includes the loss of lean body mass (LBM) which can significantly impact on response to chemotherapy and fitness for treatment, but is still largely unappreciated in clinic [14–16]. Cancer patients of lower performance status (PS) and a LBM have repeatedly been shown to have more dose limiting toxicity, a poorer chemotherapy completion rate, a higher risk of neutropenia and poorer overall survival (OS) [17–20].

Complimentary interventions such as exercise programmes aimed to improve outcomes for men on long term ADT are well documented, however robust data surrounding that for men with CRPC is lacking [21, 22]. Exercise presents as a potential effective treatment to aid in mitigating the effects of long term ADT which may be of specific benefit to this group of men, improving prostate cancer specific outcomes and LBM [22–24].

## Methods

The aim of this qualitative study was to explore the views and opinions of specialist health care professionals (HCPs) within the UK regarding prostate cancer care pathway organisation, sequencing of treatment (including fitness for treatment), the adverse-effects of treatment for men with CRPC and exercise for men with CRPC.

From December 2015 to May 2016 qualitative semi-structured interviews were undertaken with a purposively selected group of HCPs (see Table 1) responsible for prostate cancer management. HCPs were identified through national prostate cancer care teams based in the NHS or professional bodies. Seventy-eight HCPs in total were approached, and those who expressed interest (*n* = 49) were sent an invitation letter, participant information sheet and consent form via post. Once consent was obtained, dates for interview were confirmed via email or a telephone call. The interviews were digitally recorded and then anonymised. After transcription, the data were coded via Nvivo10 and analysed according to a thematic framework analysis [25].

**Table 1:** HCP demographics of those interviewed

Country of service	England	100% (37)
Profession	Consultant Urologist	24.3% (9)
	Clinical Oncologist	18.9% (7)
	Medical Oncologist	8.1% (3)
	Clinical Nurse Specialist	16.2% (6)
	General Practitioner	8.1% (3)
	Physiotherapist	8.1% (3)
	Exercise Specialist	5.4% (2)
	Service Manager	2.7% (1)
	Clinical Commissioner	8.1% (3)
	Primary Care Physician	2.7% (1)
Institution	Teaching Hospital	24.3% (9)
	District Hospital	18.9% (7)
	University	2.7% (1)
	Community	13.5% (5)
	Cancer centre	29.7% (11)
	Primary Care	10.8% (4)

Details on the interview schedule are provided in the appendix (Additional file 1). Thematic framework analysis was chosen as it was the most pragmatic approach to systematically facilitate rigorous and transparent data management without losing sight of the “raw data” and enabled the classification of the data into key themes and sub themes, judged comprehensively. This 6 step approach included familiarising with the data; generating initial codes; searching for themes; reviewing themes; devising and naming themes and producing the report [26]. This research was conducted following the guidelines for standards for reporting, process and methods from the COREQ criteria [27]. A second researcher was used to double code the interviews. A case and theme based approach was used to develop the qualitative framework.

The study protocol, topic guides and semi-structured interview schedules gained national NHS ethics approval by NRES Committee South West - Cornwall & Plymouth (15/SW/0260) and in accordance with the Governance Arrangements for Research Ethics Committees and complies fully with the Standard Operating Procedures for Research Ethics Committees in the UK. All Management permissions were sought from all NHS organisations involved in the study in accordance with NHS research governance arrangements. All participants gave written informed consent before participation in this study.

Sustained exercise TrAining for Men wIth prostate caNcer on Androgen deprivation: the STAMINA programme (RP-DG-1213-10,010). REC Reference: 15/SW/0260 IRAS Project ID: 178340 Hospital ID: STH 18391 approved on 24/08/2015.

## Results

Thirty-seven interviews with HCPs were undertaken, their demographics detailed in Table 1. Seven interviews were undertaken face to face and thirty over the telephone. Four themes were identified from the data. Verbatim quotes are provided in Table 2 to illustrate the findings.

**Table 2:** Verbatim quotes and their corresponding themes

Theme 1: The prostate cancer pathway: continuity of care	
STAMPEDE and CHAARTED	
*“Before NHS agreed to fund it [docetaxel for men with metastatic hormone sensitive disease] in January, we were just doing it based on the American study [CHAARTED], which was the more extensive group [higher volume metastatic disease] and not do the people with minimal disease. And we’re trying to still do that, just to keep the numbers down...I’m in the process of being made to say that we’re going to have to have a waiting list for these patients.”* (Medical Oncologist)	
Changes to standard of care	
*“…That has caused a problem, at an MDT, yesterday because they referred a patient who was five months out…and then the patient got upset that they weren’t offered it [docetaxel with ADT]...But then there’s no evidence for it, beyond 90 days…surgeons would argue that if there’s no evidence you should give. Whereas oncologists argue that if there’s no evidence you shouldn’t give [docetaxel].”* (Medical Oncologist)	
Theme 2: Uncertainty with treatment sequencing in CRPC	
Treatment sequencing	
“*Yes, it always has changed practice. So basically all patients who are of shall I say good performance status, have limited comorbidity are now being considered for chemotherapy alongside androgen deprivation therapy for metastatic hormone sensitive disease…So a lot of it [treatment options] is individual…[future treatment] will change somewhat because the use of chemotherapy may have happened earlier on for hormone sensitive disease.”* (Clinical Oncologist) *“But I think I probably would still go for, let’s see, I think if somebody’s had adjuvant chemotherapy when they relapse I would be more inclined to go to further hormone therapies first before going back to chemotherapy. Mm, I haven’t decided about that yet.”* (Medical Oncologist)	
Performance status, fitness for treatment and treatment decisions	
*“…to get enzalutamide or abiraterone [men with CRPC] have to be performance status zero or one. And they have to have, be asymptomatic or minimally symptomatic…So you can’t give it to patients who are poorly or you shouldn’t give it to patients who are poorly…Well actually the docetaxel performance status is zero to two. So if you have a poorly patient and some people will, if they have say liver mets or what have you, they’ll go straight to docetaxel…I would, if I had someone who was really fit, I would potentially give them enzalutamide or abiraterone pre-chemotherapy, if they had liver or lung involvement.”* (Medical Oncologist) *“…although in young fit men that probably will influence me giving docetaxel before giving abiraterone, yes, or enzalutamide…so performance status, they’d have to be PS 0 or 1 for me to give them docetaxel generally,with good renal function, and you know, just generally a good performance status” (Clinical Oncologist)* *“So you can be fairly unfit to have hormones, but for the chemotherapy we’d only offer that to people who are fit basically…at some level, able to withstand it anyway.”* (Urologist)	
Theme 3: Quality of Life and adverse effects	
Physiological adverse-effects	
*“These things go off a bit of a cliff when they start the hormone therapy, so they’ve got a sense of what they’re normally like, and they very quickly get a sense that they’re different on hormones.”* (GP) *“Fatigue, hot flushes, hot flushes are probably the top one, a change in mood...I often see men for urinary urgency and frequency.”* (Physiotherapist)	
Compromising treatment and muscle wastage	
*“Well, I think the benefits have to be twofold, don’t they, so there are disease specific benefits and then there’s QoL and they’re not necessarily aligned.”* (Urologist) *“…really quality of life is a, it’s a huge issue and there’s no point in keeping people alive if we’re wrecking their lives.”* (Clinical Oncologist) *“So I’ve seen muscle wasting that was quite significant that was stopping somebody from going out and doing their job…So, although there was data for overall survival benefit in continuing the hormones, I stopped the hormones after discussion, because I felt that we’re going to leave him housebound…”* (Clinical Oncologist) *“While I don’t have any method in clinic of assessing muscle wastage and I certainly don’t have time to sit measuring their muscle bulk...I probably should weigh them more often, but it depends what I’m going to do about it, I guess.”* (Clinical Oncologist)	
Theme 4: Prostate cancer and exercise	
NICE recommendations and purpose	
*“Well I was surprised to find out that NICE’s has actually made recommendations and usually when NICE makes a recommendation then it, it eventually happens because it means it’s going to be funded.”* (Clinical Oncologist) *“I personally think it’s a fundamental aspect of healthcare so, you know, I think it would be hugely beneficial if we had more access to it.”* (GP) *“if it was a drug, exercise would be being prescribed all the time ...”* (GP)	
Physiological and psychological benefits. *“Well I think there’s increasing evidence that exercise decreases death rate, not just prostate cancer but cardiovascular fitness and cancer, you know there is a link…*	
*So your chances of survival and good quality of life increase massively if you’ve got a normal body mass index and you’ve got cardiovascular fitness...”* (GP) *“I think an increased feeling of well-being, an increased quality of life, reduction in cardiovascular morbidity and mortality.”* (Urologist)	
Management of adverse effects	
*“…to sort of masculate them a little bit more by sort of encouraging them with exercise...and seeing the feedback that they give at the end is great really, and it’s giving them control because, you know, it’s quite a man-thing isn’t it, sort of needing to be in control a little bit more.”* (Physiotherapist) *“I think it’s beneficial for maintaining muscle strength, quality of life and exercise capacity, which I think is very important for them, and it keeps some bone strength, you know, when on their long-term hormones, the more exercise they do the more they can maintain their bone strength, which is going to be a good thing, And it’s good psychologically, you know, if they can keep going out and playing golf or doing whatever they do, then I think that’s very important for them.”* (Clinical Oncologist)	

**Theme 1: The prostate cancer care pathway: continuity of care.**

### Stampede and Chaarted

The urologists and oncologists involved in secondary care stated that the recent data from the STAMPEDE and CHAARTED trials had changed the prostate cancer pathway resulting in men with advanced hormone-sensitive disease being offered chemotherapy alongside initiation of first line ADT [9, 11]. Oncologists stated that they were facing increasing numbers of referrals of men with hormone sensitive disease and therefore had a greater role in the care pathway than prior to the pathway change, where predominantly they had treated men with CRPC. The majority felt this presented an increased workload for oncologists and potential problems that NICE or the NHS may have not foreseen, consequentially yielding further uncertainty.

### Changes to standard care

In light of the changes brought upon by STAMPEDE and CHAARTED, the pressure to change practice was felt to have put additional strain on the cross-over of patient care from urology to oncology as it ensues earlier now docetaxel is offered at hormone sensitive stages. A medical oncologist talked specifically about the time constraints surrounding the simultaneous initiation of chemotherapy and ADT. The current recommendations (based on the trial data) state that docetaxel should be initiated within 90 days of starting ADT [28].

*“[…] That has caused a problem, at an MDT, yesterday because they referred a patient who was five months out […] and then the patient got upset that they weren’t offered it [docetaxel with ADT] [...] But then there’s no evidence for it, [beyond] 90 days.”* **(Medical Oncologist).**

**Theme 2: Uncertainty with treatment sequencing in CRPC**

### Treatment sequencing

The majority of the oncologists and urologists felt that changes to the prostate cancer care pathway had resulted in dilemmas associated with the sequencing of treatment. Prior to the STAMPEDE and CHAARTED data, men with newly diagnosed CRPC would be chemo-naïve (i.e. no previous docetaxel regimen given). It was obvious amongst these HCPs that the standard of care would change for men with CRPC given that these men are likely to have had docetaxel earlier in the care pathway. Thirty-seven interviews with HCPs. 1 describes the current sequencing of docetaxel in the STAMPEDE and CHAARTED era.

*“[…] I think if somebody’s had adjuvant chemotherapy when they relapse I would be more inclined to go to further hormone therapies first before going back to chemotherapy. Mm, I haven’t decided about that yet.”* **(Medical Oncologist).**

### Performance status, fitness for treatment and treatment decisions

There were some conflicting statements regarding sequencing second line ADT (enzalutamide and abiraterone) and chemotherapy for men with CRPC. Some of the interviewees alluded to patients having to have a better PS to receive second line ADT particularly pre-chemotherapy. Other HCPs stated that men would generally have to have a better PS, or be fitter, for them to consider chemotherapy before second line ADT or at any stage.

**Theme 3: Quality of life and adverse-effects.**

### Physiological adverse-effects of treatment

Generally, adverse-effects which were commonly mentioned to be associated with ADT were fatigue, weight gain, hot flushes, muscle weakness/ wastage (particularly worse when compounded with steroids), a decrease in sex drive and breast swelling (gynecomastia). Those most commonly mentioned with chemotherapy were neutropenia (with a chronic worry of acute death), emesis (vomiting), peripheral neuropathy and fatigue.

### Impact on quality of life

The physiological effects of ADT were recognised as having a profound effect on QoL, impacting on the ability to work, social life and interpersonal relationships. In particular, effects on physical function which impaired the ability of these men to work were considered severely detrimental to QoL.

### Compromising treatment and muscle wastage

Determining the root cause of an adverse-effect is fundamental to maintaining a patient’s QoL whilst succeeding with the best possible treatment regimen to control disease. Dropping the dose, treatment breaks or switching to an alternate therapy can be an option if a man’s experience is such that the clinician regards this to be necessary.

Changes to treatment regimens were deemed necessary for some HCPs where muscle wastage becomes a problem in a patient. One example given by a clinical oncologist described how ADT was stopped due to extreme muscle wastage in one patient.

HCPs were asked if muscle wasting could be identified as a result of anti-neoplastic treatment (ADT/steroids) or the disease process (cancer cachexia). A majority felt they could adequately assess this based on a subjective assessment of the patient (by eye) and by noting any marked deterioration in PS or wellbeing over a period of time. HCPs were unanimous that currently there exists no robust diagnostic procedure when distinguishing muscle wastage of different aetiologies.

**Theme 4: Prostate cancer and exercise.**

### NICE recommendations and purpose

Almost all the HCPs seemed to have knowledge of the NICE recommendations for exercise in men with prostate cancer (section 1.4.19 in CG175). However, there was some confusion as to why, given that NICE has made the recommendations, action had not been taken nationally to implement them.

*“if it was a drug, exercise would be being prescribed all the time [ ...]”* **(GP).**

Most HCPs felt that an exercise programme had a place within healthcare, as there was perceived benefit and purpose of exercise programmes.

### Physiological and psychological benefits

Supervised exercise was viewed as having many benefits men with prostate cancer, physically and psychologically. The HCPs specifically spoke about improvements in cardiovascular health, reducing BMI, increasing muscle mass and decreasing mortality. Beneficial effects to QoL included improvements in social life, the ability work and complete activities of daily living.

### Management of adverse-effects

Some of the HCPs saw exercise as a way to manage the adverse-effects of cancer therapies and means for these men to take back some control over their health.

The physiological benefits commonly mentioned by the interviewees were the maintenance of muscle bulk and bone health, which is often compromised on ADT, and the increased tolerance of treatment and a reduction in complications (surgical or medicinal).

## Discussion

This qualitative study of 37 HCPs in the UK has highlighted a lack of continuity in the prostate cancer care pathway between urologists and oncologists and the increased workload on oncologists posed by earlier introduction of newer systemic therapies presents new challenges in optimum care for men with prostate cancer. Furthermore, uncertainty exists around optimal selection, timing and sequencing of chemotherapy and second-line treatment amongst the HCPs.

The trials which assessed the use of abiraterone and enzalutamide for men with CRPC were predominantly in men with good PS (Eastern Cooperative Oncology Group, ECOG 0–1) [5, 8, 29–31]. For the minority of men in these trials with a poorer PS (ECOG ≥2) no significant OS benefit was demonstrated with either abiraterone or enzalutamide. For this reason NICE recommends the use of these drugs in men with CRPC with no or mild symptoms. In the post-docetaxel setting abiraterone is only recommended in men whose disease has progressed on or after one docetaxel-containing chemotherapy regimen. NICE recommends use of enzalutamide in a pre-docetaxel setting or post one course of docetaxel-containing regimen in men with no or mild symptoms (PS 0–1).

The recently published LATITUDE and STAMPEDE trial data has demonstrated an OS benefit and radiographic-progression free survival benefit of abiraterone and prednisolone alongside ADT in men in men with newly diagnosed, metastatic hormone sensitive prostate cancer and men initiating long term ADT [32, 33]. It is likely that abiraterone will therefore shift to earlier use in the prostate cancer pathway, similar to the shift seen with docetaxel. There may be further uncertainty surrounding optimum therapy sequencing, and neither study assessed the initiation of ADT plus abiraterone alongside docetaxel or versus ADT plus docetaxel, so there lacks comparative data for the new standard of care. Therefore for those with a poorer PS, the need for robust data around the efficacy of subsequent treatments after progression becomes crucial.

Docetaxel is a widely used drug, relatively inexpensive and a common therapy used for CRPC. Conceptually, the move to earlier administration in the care pathway men presenting with metastatic disease simultaneous with initiation of long term ADT following publication of the CHAARTED and STAMPEDE studies [9–11] has been relatively easy. Nevertheless, it is recognised that whilst the implementation of docetaxel earlier is both recommended and feasible, there are implications on the care pathway and resource utilisation [34] with additional workload for oncologists and increased demand on oncology units. Discontinuity between urology and oncology might risk delayed referral and compromise the treatment which can be offered to a man; treatment which he should be eligible for. As described by the medical oncologist (*see* “Stampede and Chaarted” Table 2) this has arguably risked sub-optimal care by restricting the numbers of men referred for chemotherapy.

Figures from the Royal College of Radiology report estimates that 67 full-time oncology consultants are required immediately to cover the current excess of clinical workload in the NHS and nearly 1 in 5 could retire from the workforce in the next 5 years [35]. With an ever-growing cancer population which is surviving longer yet a lack of oncology work force to meet the required demands on the cancer services, the NHS is facing a potential crisis [35].

**Fig. 1:**
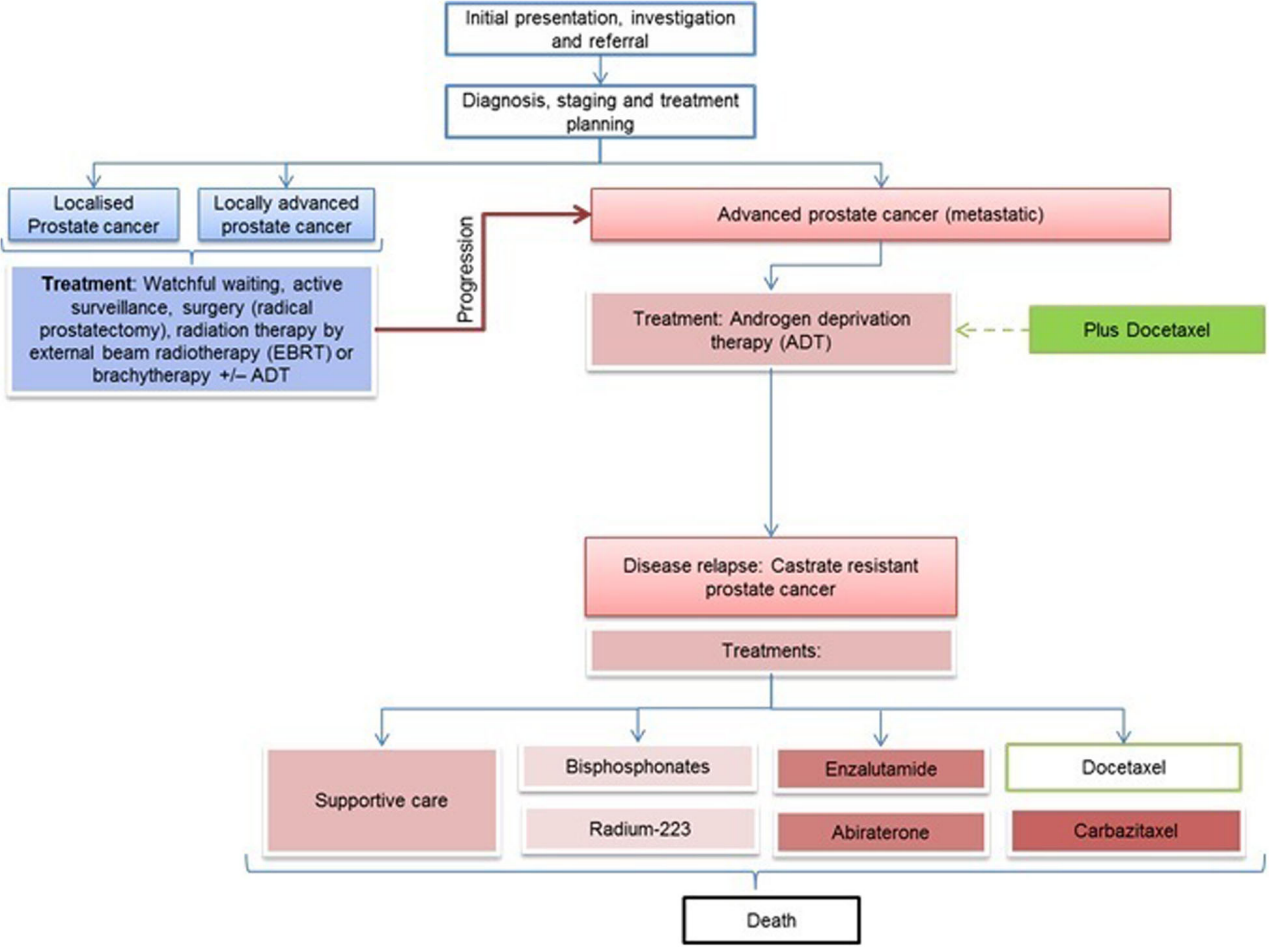
The post-STAMPEDE and CHAARTED data prostate cancer care pathway. The blue boxes represent the localised and locally advanced prostate cancer care pathway in brief. The red boxes represent the advanced prostate cancer care pathway leading to castrate resistance and the therapeutic options at this stage of disease. Steroids such as prednisone are also given as standard care alongside these drugs. The green boxes show docetaxel, now offered upon the initiation of ADT (within 90 days) at newly diagnosed hormone sensitive advanced metastatic disease (M1). This has caused uncertainty in the sequencing of treatments as the disease advances to CRPC as well as the efficacy of these drugs now men have already undergone one docetaxel regimen and are no longer “chemo-naive”. It is also unclear how a second potential docetaxel regimen would be sequenced

In addition to the immediate strain brought on by the change in the prostate cancer care pathway, it is important to consider how the introduction of docetaxel earlier will affect subsequent treatment sequencing at CRPC stages (Fig. 1). There was a lack of clarity in how docetaxel and second generation anti-androgens, abiraterone and enzalutamide, may be sequenced for men in the post-STAMPEDE era. Including how decisions would be made to give further docetaxel chemotherapy regimens, if men have previously received docetaxel, and how effective a second docetaxel regimen may be further down the line. Some of the HCPs commented that second generation anti-androgens, would be offered in the place of chemotherapy where it is felt the man may not tolerate docetaxel due to a poorer PS.

With a lack of trial data, clinical guidance and clarity surrounding treatment sequencing, treating clinicians face a major dilemma. They may have to make a treatment evaluation on a patient and potentially offer unsuitable treatments based on the premise that there is no suitable alternative. Furthermore, the optimum pre- or post-docetaxel therapy is heavily debatable in CRPC given that there is no suitable comparison data, forcing clinicians to make decisions based on assumptions and clinical experience rather than true “level one” data.

Based on the findings from the interviews, some clinicians seem to be treating patients with poorer PS’ with abiraterone preferentially over chemotherapy whilst there lacks available data as to whether it may actually improve survival. Contraindications to docetaxel use are a poor PS (ECOG 3–4, caution for those with PS 2) [36]. This gives such men even fewer treatment options, given that NICE does not recommend the use of second generation anti-androgens. With these limitations, we can conclude that fitness is a key aspect in treatment decision making by clinicians and improving or maintaining a man’s PS to 0–1 enables access to the necessary therapies and ensures the best possible outcomes.

Fitness for treatment is a predominant factor in a clinician’s treatment based decision [37] and in advanced cancer populations remains a significant barrier for access to the available therapies in patients with a poor PS. Physiological adverse-effects of ADT such as fatigue, muscle wasting and increased central adiposity, which can significantly impact on QoL, can also compromise eligibility for treatment, where it interferes with performance status.

The shift in treatment paradigms to move docetaxel earlier in the care pathway comprises of both positive effects and a degree of uncertainty when fitness for treatment is considered. On one hand, men receiving docetaxel at hormone sensitive stages will likely be on average younger and have a better PS when compared to the castrate resistant setting. The combination of docetaxel with ADT at hormone sensitive stages has also been shown to significantly increase progression free survival meaning these men enter the castrate resistant phase of the disease later [38]. These men are therefore, at this stage, not only likely to tolerate the docetaxel better, maintaining the optimum drug dosage, but prolong the time to which they will need further therapy for advancing disease. On the other hand, when men do eventually progress to CRPC, the long term effects of a previous docetaxel regimen on PS and fitness for treatment are unclear. This may also be compounded by the adverse-effects of long term ADT given that these men can remain on first line ADT for many years.

Treatment evaluation of a patient with CRPC is pertinent given the predominance of muscle wasting and deterioration in bone health [39]. The effects of muscle wastage appear to have significant implications on the fitness and PS of a man, and therefore not only impacting his current therapy but also likely to affect the future treatments offered as his disease progresses. Retrospective data has associated better OS in men with metastatic prostate cancer receiving docetaxel with increased lean body mass [20].

The findings highlighted a lack of clarity over the origin of the muscle wastage and subsequently how it may be assessed and treated, where generally the HCPs spoke of a subjective assessment “by eye”. Given that a side-effect of ADT includes central and visceral obesity; such subjective assessments are likely to be misleading [40]. This poses a significant risk specific to these men where long term ADT is likely to mask any underlying muscle wasting pathology. Equally, symptoms of muscle wastage are very generalizable and can be difficult to distinguish from that of other treatment-related side effects (e.g. fatigue, impaired immune function and metabolic abnormalities) [39]. Research must focus on accurate and objective diagnostic measures of muscle wastage to enable its successful treatment, improving both the physiological wellbeing of these men and subsequently their response to cancer therapies.

There was an overwhelming view amongst HCPs that currently very little is offered in the way of treatment to address muscle wastage. Generally, diet and exercise advice was offered for a majority of muscle wastage seen in the clinic. Success from this approach was viewed as variable and may be in part due to a lack of consistency from HCP to HCP in the subjective nature of general “exercise and diet advice” and the “one size fits all” approach.

Compromising treatment was also mentioned by some of the HCPs. Cessation of ADT or restricting the use of steroids may be the case for men where muscle wastage is of a significant detriment to QoL at the potential cost of a survival benefit.

The consensus amongst the HCPs was that exercise presents as an effective therapy, improving both physiological and psychological outcomes as well as a tool aiding in the management of adverse-effects. Almost all the HCPs seemed to have knowledge of the current NICE recommendations however there was some confusion as to why action had not been taken nationally to implement them.

As described earlier, it is clear that maintaining or improving the PS of a man through his prostate cancer journey is critical to obtaining the best possible outcomes. This includes the potential alleviation of adverse-effects of treatment and the maintenance of a good QoL, where the two go hand in hand. There is increasing evidence demonstrating that exercise may represent a useful stand alone or combination therapy for the treatment of cancer, improving physiological and psychosocial outcomes [41]. In addition, specific beneficial effects of exercise training for improving lean body mass (LBM) are also well established [24, 42].

By improving physical fitness through exercise there is potential to not only improving the chances of receiving, but also better tolerating, the appropriate cancer treatments. Studies investigating the effectiveness of resistance and aerobic training in cancer populations have demonstrated an increase in chemotherapy completion rate and treatment toxicities [42–44].

Most of the HCPs felt that exercise should form a fundamental part of healthcare throughout the prostate cancer care pathway. Support should be offered from the beginning of a patient’s journey with prostate cancer and carried right to the end even where he may reach the castrate resistant phase of the disease, although there is a significant lack of data for such interventions in the population.

It is important to acknowledge the limitations to this study. This qualitative study did not seek to establish generalizability of findings but sought to gain a deeper insight into the views and opinions of a selected group of HCPs regarding the prostate cancer care pathway. The study has highlighted some critical issues facing prostate cancer treatment and management within the NHS. However it is acknowledged that testing these findings among a wider population of HCPs would be is warranted in order to test the generalizability of the findings. As the majority of interviewees were urologists and oncologists the data may be more biased to the perspectives of this particular group of professionals. Due to the nature of how these participants were recruited into the study the authors acknowledge that a self-selection bias may also exist as 63% (*n* = 49) of the HCPs approached expressed an interest in the research themes; the sampling of the participants in this study failed to address the views of those who did not express an interest. The thematic framework approach to analysing the data were used, although commonly used in healthcare research; this form of analysis is more inductive and therefore stays strongly informed by a priori reasoning [45].

To our knowledge, this is the first qualitative study of HCPs to have focused on second-line treatment sequencing; changes to practice due to the STAMPEDE and CHAARTED trial data; adverse-effects of prostate cancer treatment including muscle wastage, compromising treatment and finally prostate cancer and exercise, with a focus to men with CRPC. This study has highlighted the need to investigate further with a wider group of HCPs as well as involving the views, opinions and experiences of men with CRPC. Further observations are needed to develop clarity in the current prostate cancer care pathway, identifying weaknesses as we evolve and refine how we treat prostate cancer. Efforts need to be made to help expand the oncology workforce as the demand for cancer care is ever increasing. This will help enable clinicians to carry out consistent care but also recognise the need to vary treatment regimens dependant on a patient’s individual needs. This is particularly the case for those with CRPC who may have remained on ADT for a number of years and therefore experience significant detrimental adverse-effects from treatment including muscle wastage. In addition, this study has highlighted a lack suitable exercise provision based on the NICE recommendations. Future research should focus on how this can be improved and particularly in men with more advanced disease who have a higher disease burden, where the current data for exercise in this population lacks.

## Conclusions

The prostate cancer care pathway, including the optimum sequencing of drugs, is evolving and further work will be needed to reassess and optimize this pathway in light of its recent changes. The adverse-effects of prostate cancer treatments have a significant detrimental effect to patient QoL. Exercise may present as a useful stand alone or combination therapy in both the alleviation of adverse-effects of treatment but also of the tolerance to treatment, particularly where programmes aim to increase LBM. In addition, fully integrated exercise programmes may enable these men to retain or improve their PS ensuring access to all available treatment options. Such programmes should be available throughout the prostate cancer care pathway and more research is needed for those at more advanced stages of disease, particularly in CRPC where data are lacking. A highly functioning, refined prostate cancer care pathway with integrated exercise programmes will allow men to maximise the benefits of the many treatments they may have but also live well during this period, maintaining a good QoL.

## Additional file

Additional file 1: Supplementary material v1. Interview schedules. The schedules covered exercise programmes for men with prostate cancer; second-line treatment sequencing; changes to practice due to the STAMPEDE and CHAARTED trial data; the HCPs role in the current care pathway for men with prostate cancer and finally muscle loss in CRPC. Two interview schedules were used; the second interview schedule was an amended version of the first to contain questions regarding the recent changes to clinical practice due to the STAMPEDE and CHAARTED trial data and the care for men with CRPC. This schedule specifically sought the views of the HCPs directly involved in the treatment planning for men with prostate cancer (urologists and oncologists). The schedule 1 consisted of 16 questions and schedule 2 contained 18 questions.

## Abbreviations

ADT: Androgen deprivation therapy
BMI: body mass index
COREQ: Consolidated criteria for reporting qualitative research
CRPC: Castrate resistant prostate cancer
ECOG: Eastern Cooperative Oncology Group
HCP: Health care professionals
LBM: Lean body mass
NHS: National Health Service
NICE: National institute of clinical excellence
OS: Overall survival
PS: performance status
QoL: Quality of Life
