# Supplementary material for: Treatment in the STAMPEDE era for castrate resistant prostate cancer in the UK: ongoing challenges and underappreciated clinical problems
Source: BMC Cancer. 2018 Jun 19;18:667. doi: 10.1186/s12885-018-4527-y (PMC6006691; doi:10.1186/s12885-018-4527-y)
Supplement: Supplementary file 1 — Supplementary material v1. Interview schedules. The schedules covered exercise programmes for men with prostate cancer; second-line treatment sequencing; changes to practice due to the STAMPEDE and CHAARTED trial data; the HCPs role in the current care pathway for men with prostate cancer and finally muscle loss in CRPC. Two interview schedules were used; the second interview schedule was an amended version of the first to contain questions regarding the recent changes to clinical practice due to the STAMPEDE and CHAARTED trial data and the care for men with CRPC. This schedule specifically sought the views of the HCPs directly involved in the treatment planning for men with prostate cancer (urologists and oncologists). The schedule 1 consisted of 16 questions and schedule 2 contained 18 questions. [file 12885_2018_4527_MOESM1_ESM.docx]

**Treatment in the STAMPEDE era for castrate resistant prostate cancer in the UK: ongoing challenges and underappreciated clinical problems**

Authors: Rosa U Greasley^1^, Rebecca Turner^2^, Karen Collins^3^, Janet Brown^4^, Liam Bourke^1†*^, Derek J Rosario^2†^

**Supplementary material**

**Interview schedules**

The schedules covered exercise programmes for men with prostate cancer; second-line treatment sequencing; changes to practice due to the STAMPEDE and CHAARTED trial data; the HCPs role in the current care pathway for men with prostate cancer and finally muscle loss in CRPC. Two interview schedules were used, the second interview schedule was an amended version of the first to contain questions regarding the recent changes to clinical practice due to the STAMPEDE and CHAARTED trial data and the care for men with CRPC. This schedule specifically sought the views of the HCPs directly involved in the treatment planning for men with prostate cancer (urologists and oncologists). The schedule 1 consisted of 16 questions and schedule 2 contained 18 questions.

**Interview schedule 1**

#### Introduction

Thank you for your time in taking part in this interview. We are interested in your perspective regarding roles, responsibilities and training needs associated with providing supervised exercise programmes for men with prostate cancer on ADT. By supervised exercise, we mean a structured programme of exercise training delivered and overseen by a professional.

We would like to audio record the interviews but these will be completely confidential and all data will be anonymised in transcription and analysis. Can you please confirm you have read, understood and signed the informed consent form and are happy to proceed?

#### Questions

- Can you tell me a little about your current role in the care pathway for men with prostate cancer on androgen deprivation therapy (ADT)?
- How do your patients cope with their cancer and ADT?
  - What are the common adverse-effects with this treatment, and which do you feel men find most bothersome?
  - How would you say being on ADT for prostate cancer affects men’s quality of life?
  - Are you aware of any non-pharmacological treatment or any supportive programmes designed to improve quality of life for men on ADT?
- What do you know about the role of exercise in treating men with PC? (knowledge)
  - Are you aware of any guidance? Can you tell me what the NICE recommendations are as you see them? (knowledge)
  - How do you feel about behaviour change strategies like looking at worries and concerns and setting goals? Do you think they have a role in exercise programmes? (beliefs about consequences)

Current guidance recommends men with prostate cancer on ADT have access to supervised exercise which should include an exercise prescription as well as behavioural support such as goal setting and addressing worries and concerns.

- How do you feel about this:
  - As part of standard NHS practice?
  - Should this be separate from NHS care?
- What is your organisation already doing with regards to exercise for men with prostate cancer? (memory, attention, decision)
- Are you currently involved in setting goals with your patients and do you follow up on whether these are achieved or not?
- Are you aware of any exercise programmes for other patient groups? How beneficial do you think exercise/exercise programmes would be for your patients with prostate cancer?
- Who’s role would you see it as to i) make referrals for exercise programmes ii) delivery of exercise? (social/professional role identity)
  - Should this take place in primary/secondary care/community/outpatient settings (who specifically?)

*Probe* who should introduce idea to patient, delivering exercise (prescription & behaviour change elements), following up with patients the amount of exercise being done. If you do not see it as your role, can you elaborate as to why and who might be better placed?

- Have you been involved in referral or delivery of exercise programmes to any other patient groups in the past?

Our research team are hoping to evaluate how a 12 week, or 12 month, supervised exercise programme could be delivered in the NHS for men on ADT. This will require professionals in your role to support this process. That might involve making referrals, delivering the exercise programme and providing specialised behaviour change support. How would you feel if one or more of these elements became part of your role? (social/professional role identity, emotion)

- - What applicable skills do you think you currently have? Do you think you’d be able to new relevant learn skills (what training would that need) (skills)
  - Given training do you think you’d feel confident in doing this? (optimism)
  - How difficult or easy do you think it would be for you to do? (beliefs about capabilities)
  - Would it be something you’d like to do (all/part/none)? (goals)
  - Would it be compatible with how you see your role? (social/professional role identity)
  - How do you think your colleagues/seniors e.g. consultants/managers would respond, would this help or be a problem? (social influences)
  - Would there be capacity to support a 12 week or 12 month programme? What would help to facilitate capacity? (environmental context and resources)
- Given people were trained and happy to do this what barriers might there be to putting it in place from your point for view? Does this differ between a 12 week and 12 month commitment?(environmental context and resources)
  - Practical/resource
  - From staff, patients, systems?
  - What do you think would happen if you did take this on? (for self/patients)
  - If this did become part of your role is there anything which would make it more likely you’d do it (incentives), what would these look like? (Reinforcement)
  - Would there be systems that could help monitor if it’s being done, make it easier? (Behavioural regulation)
- If the exercise programme was put in place what do you think patients would think about it?
  - Positive/negative reactions
  - Barriers to attending,
  - What would make them more likely to attend and maintain their involvement?
- Going back to training, if this was to take place, would you be able to undertake training sessions in your current role?
- What would be the best way to deliver the training?

*Probe* format, length, time of day, location, number of sessions, duration of sessions, practical element with supervision, including videoing?

- Would you be prepared to do things like homework, keep a reflective journal?
- If we were to develop a training programme with a view to implementing an intervention would you be interested in taking part? (Intention)

- If not, why not?

**Interview schedule 2**

Introduction

Thank you for your time in taking part in this interview. We are interested in your perspective regarding roles, responsibilities and training needs associated with providing supervised exercise programmes for men with metastatic castrate resistant prostate cancer (mCRPC). By supervised exercise, we mean a structured programme of exercise training delivered and overseen by a professional. We are also interested to establish the views and opinions of such interventions with the aim to improve outcomes of a structured exercise programme.

We would like to audio record the interviews but these will be completely confidential and all data will be anonymised in transcription and analysis. Can you please confirm you have read, understood and signed the informed consent form and are happy to proceed?

Questions

STAMPEDE trial data

- The standard of care for advanced hormone sensitive prostate cancer is long term-androgen deprivation therapy. How much do you agree with this statement?
- Recent data from the STAMPEDE and CHAARTED trial suggest there to be a survival benefit in initiating chemotherapy earlier in the hormone sensitive advanced PCa pathway. Do you feel the recent findings of the trials will change the standard of care, and to what extent?

**[PROBE]**

- How might you change your own practice?

The HCPs role and current pathway for men with metastatic castrate resistant prostate cancer (mCRPC)

- What is your role within the care pathway for men whose cancer has relapsed (i.e. become castrate resistant)?

**[PROBE]**

- Involved in the treatment of these men: How do you typically sequence treatment for men with mCRPC? [Chemotherapy first? 2nd line ADT first? Other?]
- Will this change based on the STAMPEDE and CHAARTED trial data?
- For these men (mCRPC), what are the most common reasons that effect not only the initiation of 2^nd^ line treatment but also the duration?

**[PROBE]**

- Fitness - How might you assess these men for fitness to initiate 2^nd^ line treatment and what specifically might you find that would prevent you in prescribing such treatment?
- Impact on QoL - What specifically may result in a poorer QoL?
- Clinician's advice - What specifically may influence the clinician?
- In your experience what do you consider to be the most important outcome for men with mCRPC?
- What supportive and/or palliative programmes for men with mCRPC do you know of?

**[PROBE]**

- Would you refer routinely into such programmes and if so what factors might prompt you to?
- Local / National?
- In your opinion how successful have they been?

Muscle loss and cachexia in mCRPC

- In your experience, what adverse effects do you consider to have the most impact on men with mCRPC?

**[PROBE]**

- Treatment specific?
- Disease specific?
- What impact does muscle wastage have on these men?

**[PROBE]**

- Do you consider it to be clinically important?
- What do you do currently to address muscle wastage in men with mCRPC?

**[PROBE]**

- Do you consider the cause of muscle wastage? (do you distinguish between muscle wastage associated with ADT and inactivity or cachexia and sarcopenia) - is there any merit to that?
- How do you assess?
- What treatments might you implement?
- How successful have you found these? Adverse effects?
- What might prompt you to initiate such treatments?
- Are there any barriers to addressing muscle wastage?
- Are there any specific therapies you might offer for a man with mCRPC with suspected cachexia or early onset cachexia? (different to treatment strategies for muscle wastage)

**[PROBE]**

- What therapies?
- How successful have you found these therapies?

Prostate cancer and exercise interventions

- What do you know about the role of exercise in treating men with PCa?

**[PROBE]**

- Could you describe any guidance or recommendations you are aware of for these men?
- What is your organisation already doing with regards to exercise for men with prostate cancer on ADT?
- How beneficial do you think exercise/exercise programmes would be for your patients with mCRPC?

**[PROBE]**

- Would you be prepared to directly advocate and be personally involved in exercise programmes for men in your clinics?
- Where do you think exercise should fit in the treatment pathway for men with mCRPC? (Before initiation of chemotherapy/2nd line ADT, during or after?)
- Are there any additional behavioural change strategies you feel might complement exercise programmes?
- Which health care professional do you feel should be responsible for referring and following up exercise interventions in men with mCRPC?

**[PROMPT]** Urologist/Oncologist/GP/other?

- What are the barriers you foresee for men with mCRPC in enrolling in a 12 week exercise programme?

**[PROBE]**

- - Practical/resource (Is there currently capacity?)
  - From staff, patients, systems?
  - Patient related personal barriers?

**Our research team are hoping to evaluate how a 12 week supervised exercise programme, potentially in combination with a pharmaceutical agent to improve response, can be delivered in the NHS for men initiating 2^nd^ line treatment for mCRPC. This will require professionals in your role to support this process.**

- How would you feel about referring your mCRPC men to a study which would investigate an exercise intervention?
- Given what we have spoken about today, how would you move forward to improve outcomes in men with mCRPC?

**[PROBE]**

- What would be the best approach?
- Where should research be focussed?
- Is there anything else you would like to add?
